# Development of Spike Receptor-Binding Domain Nanoparticles as a Vaccine Candidate against SARS-CoV-2 Infection in Ferrets

**DOI:** 10.1128/mBio.00230-21

**Published:** 2021-03-02

**Authors:** Young-Il Kim, Dokyun Kim, Kwang-Min Yu, Hogyu David Seo, Shin-Ae Lee, Mark Anthony B. Casel, Seung-Gyu Jang, Stephanie Kim, WooRam Jung, Chih-Jen Lai, Young Ki Choi, Jae U. Jung

**Affiliations:** a College of Medicine, Chungbuk National University, Cheongju, Republic of Korea; b Medical Research Institute, Chungbuk National University, Cheongju, Republic of Korea; c Zoonotic Infectious Disease Research Center, Chungbuk National University, Cheongju, Republic of Korea; d Department of Cancer Biology, Lerner Research Institute, Cleveland Clinic, Cleveland, Ohio, USA; e Global Center for Pathogen Research and Human Health, Lerner Research Institute, Cleveland Clinic, Cleveland, Ohio, USA; Icahn School of Medicine at Mount Sinai

**Keywords:** coronavirus, ferret, ferritin, immunization, nanoparticle, receptor-binding domain, vaccines

## Abstract

Severe acute respiratory syndrome coronavirus 2 (SARS-CoV-2), a causative agent of the CoV disease 2019 (COVID-19) pandemic, enters host cells via the interaction of its receptor-binding domain (RBD) of the spike protein with host angiotensin-converting enzyme 2 (ACE2). Therefore, the RBD is a promising vaccine target to induce protective immunity against SARS-CoV-2 infection. In this study, we report the development of an RBD protein-based vaccine candidate against SARS-CoV-2 using self-assembling Helicobacter pylori-bullfrog ferritin nanoparticles as an antigen delivery system. RBD-ferritin protein purified from mammalian cells efficiently assembled into 24-mer nanoparticles. Sixteen- to 20-month-old ferrets were vaccinated with RBD-ferritin nanoparticles (RBD nanoparticles) by intramuscular or intranasal inoculation. All vaccinated ferrets with RBD nanoparticles produced potent neutralizing antibodies against SARS-CoV-2. Strikingly, vaccinated ferrets demonstrated efficient protection from SARS-CoV-2 challenge, showing no fever, body weight loss, or clinical symptoms. Furthermore, vaccinated ferrets showed rapid clearance of infectious virus in nasal washes and lungs as well as of viral RNA in respiratory organs. This study demonstrates that spike RBD-nanoparticles are an effective protein vaccine candidate against SARS-CoV-2.

## Abstract (precis)

Severe acute respiratory syndrome coronavirus 2 (SARS-CoV-2), a causative agent of the CoV disease 2019 (COVID-19) pandemic, enters host cells via the interaction of its receptor-binding domain (RBD) of the spike protein with host angiotensin-converting enzyme 2 (ACE2). Therefore, the RBD is a promising vaccine target to induce protective immunity against SARS-CoV-2 infection.

## INTRODUCTION

Severe acute respiratory syndrome coronavirus 2 (SARS-CoV-2), originally named the 2019 novel CoV (2019-nCoV) upon initial isolation from Wuhan, China, in December 2019, has caused a global outbreak of coronavirus disease 2019 (COVID-19), with significant socioeconomic impacts (1, 2). From the continuously growing numbers of diagnoses and deaths, COVID-19 was declared a public health emergency of international concern (PHEIC) in January 2020 and soon declared a pandemic by the WHO in March 2020 (3, 4). As of 27 January 2021, more than 100 million people have been infected with SARS-CoV-2, among which 2 million have died (5). Although approximately 80% of the patients with confirmed SARS-CoV-2 infections are asymptomatic or show mild flu-like symptoms, 20% of them progress to severe pneumonia and acute respiratory distress syndrome requiring hospitalization and mechanical ventilation (6, 7). The overwhelming number of SARS-CoV-2 patients has rapidly devastated the availability of health care resources (8). A shortage of medical resources and staff in conjunction with the overwhelming number of patients have exacerbated the quality of medical care and eventually increased the mortality rates of COVID-19 (9). Although a significant proportion of the infected patients have recovered, many of them report cardiovascular, pulmonary, and neurologic symptoms lasting after the recovery (10, 11). Thus, strong preventive measures are essential to halt the pandemic and its destructive effects on global public health, as well as the economy.

SARS-CoV-2 is a member of the *Coronaviridae* family, carrying a single positive-stranded RNA genome within the viral envelope (2). Although at least seven coronaviruses are known as etiological agents of mild respiratory illnesses in human infection, the family had not been closely associated with severe illnesses until the relatively recent outbreaks of SARS-CoV, Middle East respiratory syndrome CoV (MERS-CoV), and SARS-CoV-2 (1, 12). The emergence of these pathogens and the COVID-19 pandemic have called for urgent global research efforts to investigate the pathogenesis of coronaviruses. The SARS-CoV-2 RNA genome is approximately 30 kb and encodes structural proteins, such as spike (S), envelope (E), membrane (M), and nucleocapsid (N), and nonstructural proteins, such as papain-like protease, chymotrypsin-like protease, and RNA-dependent RNA polymerase (13). The heavily glycosylated S protein protruding from the virion surface is the key bridge between the virus and the host cell, playing a crucial role in host cell receptor recognition, virion attachment, and ultimately entry into the host cell. S is a member of the class I viral fusion protein, which undergoes trimerization upon cleavage into the S1 and S2 domains by a host cellular protease, furin. While S1 confers specificity in cell tropism through its receptor-binding domain (RBD), which directly interacts with the receptor of SARS-CoV-2, angiotensin-converting enzyme 2 (ACE2), S2 mediates membrane fusion via formation of a trimeric hairpin structure from its heptad repeat domains (14). Therefore, the S1 RBD has been considered one of the most promising candidates in vaccine development to protect against coronaviruses (15–17). Its efficacy has previously been shown to induce potent neutralizing antibodies against MERS-CoV (18). Furthermore, previous studies of neutralizing antibodies from naturally recovered patients of SARS-CoV-2 infections have mapped their epitopes to be S1 and the RBD (19, 20), implicating RBD-targeting antibodies in successful immunity against SARS-CoV-2 (21–23). Thus, most of the currently developed vaccines against SARS-CoV-2, despite their diversity in vaccine approaches, include the RBD in their immunogens (24–28).

One major limitation of small soluble proteins alone as vaccine candidates is that our immune system reacts efficiently only against immunogens of nanometer range in size (29, 30). Therefore, many protein vaccines using viral proteins are developed into virus-like particles (VLPs), which are multiprotein structures that mimic the organization and conformation of native viruses but lack the viral genome. However, this approach is limited to a few pathogens that are capable of self-assembling into VLPs upon overexpression of the viral protein, such as the hepatitis B virus (HBV) surface antigen (HBsAg) and human papillomavirus (HPV) L1 protein (31–33). Fortunately, the latest advances in molecular biology and nanotechnology have overcome this limitation by adopting nanoparticle engineering to serve as a platform for vaccines. The efficacy of these nanoparticle-engineered vaccines exceeds that of traditional vaccines, such as whole inactivated vaccines of bacterial and viral pathogens (34–38). Moreover, recent studies have shed light on the immunological advantages of nanoparticle-based vaccines in nearly every step of humoral and cellular immunity: efficient antigen transport to draining lymph nodes and antigen presentation by follicular dendritic and helper T cells, as well as high levels of activation of the germinal centers (30, 39, 40). Among the genetically engineered nanoparticles, ferritin is the most well characterized in the bionanotechnology field. Ferritin, ubiquitous through kingdoms of life, has a conserved role in minimizing damage to cells from reactive oxygen species formed from the Fenton reaction upon exposure to excess iron(II). Due to its natural tendency to self-assemble into 24-meric homopolymer and amenability via fusion peptides, ferritin is an ideal candidate for drug delivery and vaccine development (41, 42). Most importantly, its exceptional chemical and thermal stability does not require stringent temperature control, enabling a streamlined distribution process, especially in areas with limited resources for cold-chain supplies (41, 43). One of the recently engineered ferritins for vaccine development is the self-assembling Helicobacter pylori*-*bullfrog (*Rana catesbeiana*) hybrid ferritin which carries NH_2_-terminal residues from the lower subunit of bullfrog ferritin on the core of Helicobacter pylori ferritin to form radially projecting tails (38). The H. pylori ferritin-based nanoparticle has been reported to be an effective platform for vaccines to carry trimeric glycoproteins for presenting viral immunogens on its 3-fold axis points. Most importantly, it provides stronger protective immunity at a lower dose than soluble immunogens against influenza and Epstein-Barr viruses, while minimizing the risk of autoimmunity through its genetic diversity from the heavy and light chains of human ferritin (38, 44, 45).

Despite recent efforts to develop mouse models that fully recapitulate human SARS-CoV-2 infection, the current human ACE2 (hACE2)-transgenic mouse model fails to mimic pathogenic progress and symptoms of COVID-19 in humans. Ferrets (Mustela putorius furo), on the other hand, are naturally susceptible to human respiratory viruses, e.g., respiratory syncytial virus (46), influenza virus (47, 48), and SARS-CoV (49, 50), making ferret models ideal to study respiratory virus infections in humans. In addition, ferrets share with humans the anatomy of upper and lower respiratory tracts, the architecture of terminal bronchioles, and the density of submucosal glands (51, 52). Recently, we and others have shown that SARS-CoV-2-infected ferrets develop immune responses and pathogenic progress similar to humans’ and shed virus through nasal wash, saliva, urine, and fecal samples, which highly recapitulates human SARS-CoV-2 infection (53–56). Furthermore, we have also demonstrated the efficacy of the ferret model in drug discovery for SARS-CoV-2 (57). Thus, ferrets represent an infection and transmission animal model of SARS-CoV-2 that should facilitate the development of SARS-CoV-2 therapeutics and vaccines.

Here, we demonstrate the immunogenic efficacy of the self-assembling spike RBD-ferritin nanoparticle (RBD-nanoparticle) as an efficient SARS-CoV-2 vaccine antigen. We purified the RBD-nanoparticle from transfected HEK293T cells and immunized ferrets via the intramuscular (i.m.) and intranasal (i.n.) routes to monitor the induction of neutralizing antibodies. Furthermore, we challenged the vaccinated ferrets with SARS-CoV-2 and observed protective immunity against SARS-CoV-2. We propose the self-assembling RBD-nanoparticles as a potential vaccine candidate that effectively protects against SARS-CoV-2 infection.

## RESULTS

### Purification and characterization of RBD-ferritin nanoparticles.

**FIG 1:**
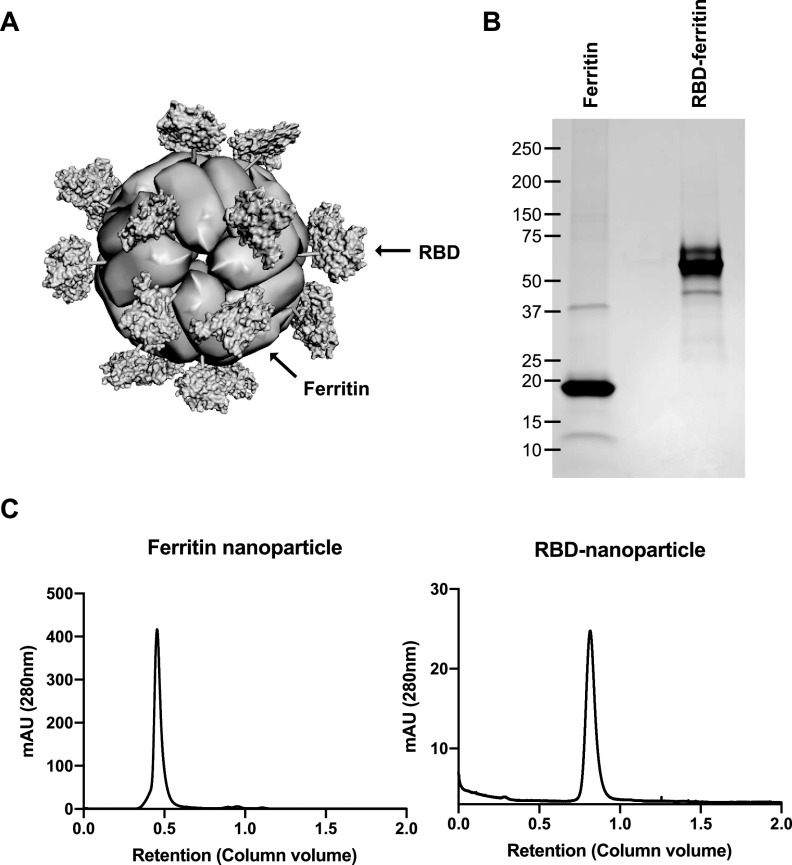
Design and purification of RBD-nanoparticles. (A) Computer-assisted modeling of an RBD-nanoparticle based on previously solved structures of H. pylori ferritin (PDB accession no. 3EGM) and SARS-CoV-2 RBD (PDB accession no. 7JMP). The RBD forms radial projections on a 3-fold axis point of the fully assembled nanoparticle. (B) Coomassie blue staining of purified ferritin-nanoparticles and RBD-nanoparticles following SDS-PAGE. (C) Size exclusion chromatography peaks of the concentrated supernatants from HEK293T cells transfected with plasmids encoding secreted ferritin-nanoparticles or RBD-nanoparticles. The supernatants were concentrated with 100-kDa-MWCO and 500-kDa-MWCO filters on a TFF system and loaded onto Superdex 200 Increase 10/300 GL and HiPrep 16/60 Sephacryl S-500 HR gel filtration columns on a Bio-Rad NGC chromatography system, respectively. mAU, arbitrary units (in thousands).

Kanekiyo et al. have discovered the use of engineered ferritin in vaccine developments by fusing it with viral immunogens (38, 44). Briefly, the NH_2_-terminal tail from the lower subunit of bullfrog ferritin was fused to H. pylori ferritin so that the bullfrog-originated tail and viral immunogen were fused by the linker and presented on the 3-fold axis points of the H. pylori ferritin core. The human codon-optimized RBD of SARS-CoV-2 Wuhan-Hu-1 strain (NCBI accession no. NC_045512) was fused to the interleukin 2 (IL-2) signal peptide at the amino terminus and the H. pylori-bullfrog ferritin at the carboxyl terminus to generate the RBD-ferritin fusion. Computer-assisted modeling predicts the three-dimensional structure of RBD-ferritin nanoparticles, with RBD forming radial projections on the 3-fold axis point of fully assembled nanoparticles (Fig. 1A). Ferritin and RBD-ferritin fusion proteins were readily purified from the supernatants of transfected HEK293T cells (Fig. 1B). To demonstrate the 24-mer self-assembly of ferritin nanoparticles, purified ferritin and RBD-ferritin proteins were subjected to size exclusion chromatography, with columns designed to have a maximum resolution for proteins with kilodalton and megadalton ranges of molecular weight. As a result, the purified ferritin nanoparticles and RBD-nanoparticles showed peaks at approximately 408 kDa and 1,350 kDa, respectively, corresponding to a 24-mer of each protein (Fig. 1C). These results indicate that RBD-ferritin protein is readily purified from mammalian cells to homogeneity and efficiently assembles into 24-mer nanoparticles.

### Immunization with RBD-nanoparticle induces neutralizing antibody in ferrets.

**FIG 2:**
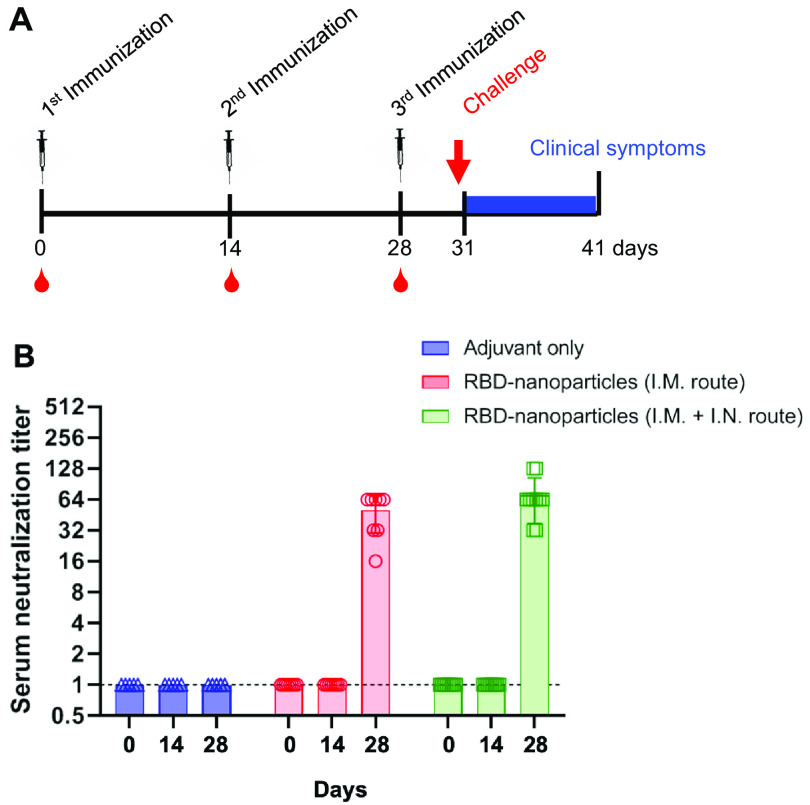
Immunization with RBD-nanoparticles elicits neutralizing antibody formation. (A) Immunization schedule of ferrets. At day 31, ferrets were challenged with 10^5.0^ TCID_50_/ml of SARS-CoV-2 and observed for clinical symptoms for the following 10 days. One group was immunized with only PBS and adjuvant (adjuvant only), and two other groups were immunized with 15 μg RBD-nanoparticles in adjuvant at a 1:1 ratio for a total volume of 600 μl. (B) Serum neutralization titers of adjuvant-immunized, RBD-nanoparticle i.m. immunized, or RBD-nanoparticle i.m. and i.n. immunized ferrets. Neutralizing antibody titers against SARS-CoV-2 NMC2019-nCoV02 (100 TCID_50_) of ferritin-nanoparticle-immunized groups were measured in Vero cells with serially diluted ferret sera collected before immunizations at days 0, 14, and 28.

To test the vaccine efficacy of purified RBD-nanoparticles, we immunized ferrets that were 16 to 20 months old (*n* = 10/immunization route), which is equivalent to 30 years of age in humans. While intramuscular (i.m.) immunization is the most widely used route for vaccine delivery, intranasal (i.n.) immunization closely resembles infection with respiratory pathogens and efficiently stimulates mucosal immunity (58). Ferrets were injected with 15 μg RBD-nanoparticles via the i.m. route only or via both the i.m. and i.n. routes over 31 days, with boosting immunizations at days 14 and 28 (Fig. 2A). Blood was drawn from each ferret prior to primary and boosting immunizations on days 14 and 28. All ferrets vaccinated with RBD-nanoparticles produced strong neutralizing antibodies after the second boosting immunization, performed at day 28. Neutralization titers did not show statistically significant differences between the routes of immunization (Fig. 2B). These data indicate that RBD-nanoparticle immunization induces strong neutralizing antibody regardless of the route of immunization.

### Immunization with RBD-nanoparticles promotes rapid viral clearance and protects ferrets from SARS-CoV-2 challenge.

**FIG 3:**
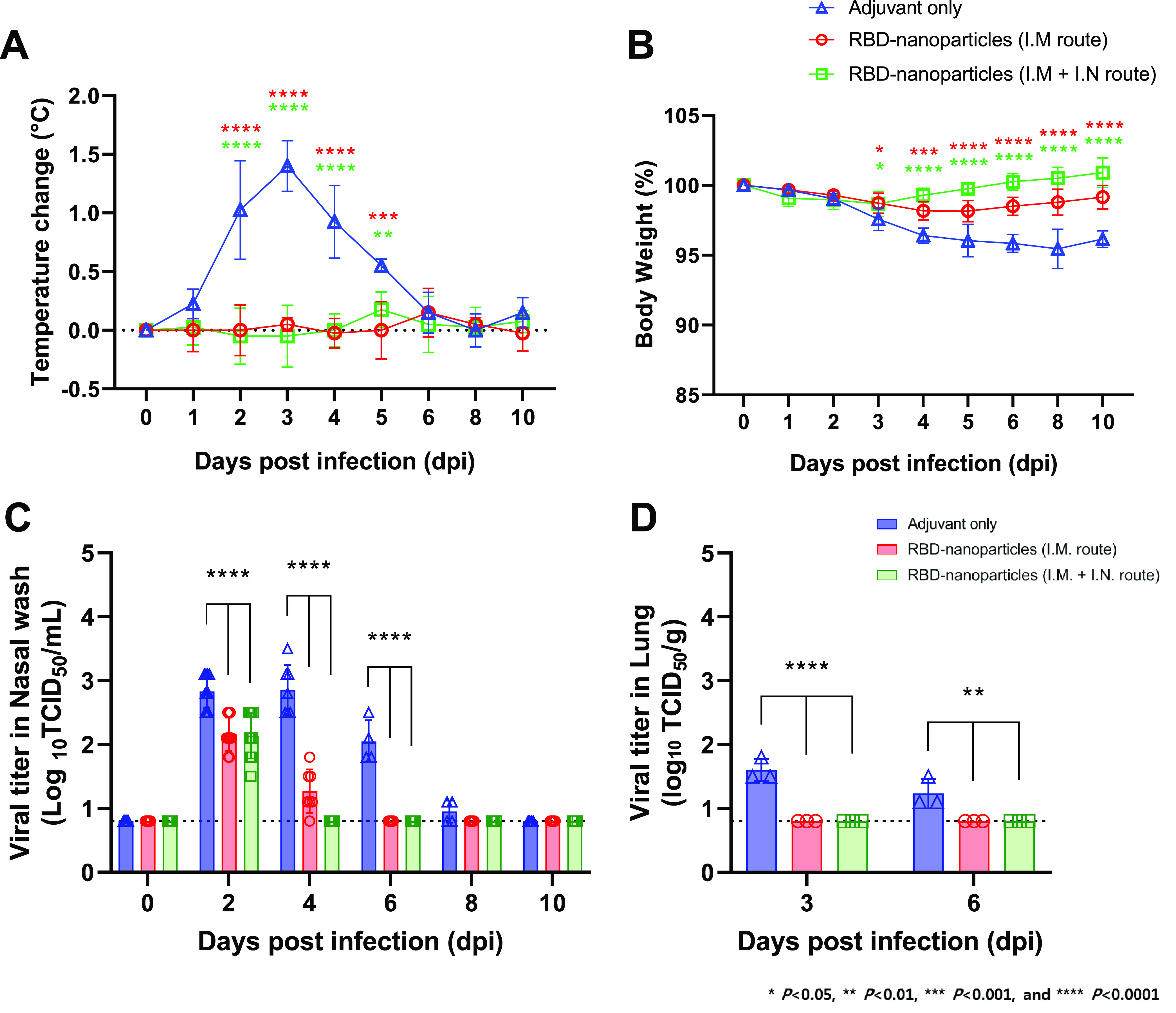
Immunization with RBD-nanoparticles promotes rapid viral clearance and protects ferrets from SARS-CoV-2 challenge. (A) Body temperature changes of adjuvant-immunized, RBD-nanoparticle i.m. immunized, or RBD-nanoparticle i.m. and i.n. immunized ferrets upon SARS-CoV-2 challenge. (B) Body weight changes of adjuvant-immunized, RBD-nanoparticle i.m. immunized, or RBD-nanoparticle i.m. and i.n. immunized ferrets upon SARS-CoV-2 challenge. (C) Viral titer in the nasal washes of adjuvant-immunized, RBD-nanoparticle i.m. immunized, or RBD-nanoparticle i.m. and i.n. immunized ferrets upon SARS-CoV-2 challenge. (D) Viral titer in the lung tissue homogenates of adjuvant-immunized, RBD-nanoparticle i.m. immunized, or RBD-nanoparticle i.m. and i.n. immunized ferrets upon SARS-CoV-2 challenge.

Immunized ferrets were challenged with 10^5.0^ 50% tissue culture infective doses (TCID_50_)/ml of NMC2019-nCoV02 strain SARS-CoV-2 3 days after the last immunization at day 31 and were monitored for clinical symptoms resembling COVID-19. Ferrets with adjuvant-only immunization were included as a control group. Over a total of 10 days from the day of challenge infection, ferrets with adjuvant-only immunization showed an increase in body temperature and a decrease in body weight (Fig. 3A). In contrast, ferrets immunized with RBD-nanoparticles did not show any change in either body temperature or body weight (Fig. 3A and B). Minor body weight changes in ferrets immunized by the i.m. route showed a statistically insignificant difference from the body weight changes of the adjuvant-only-immunized ferrets (Fig. 3B). On the other hand, ferrets immunized by the i.m. and i.n. routes provided stronger protection, with high statistical significance against body weight loss, as shown by the minimal reduction of body weight followed by a constant increase thereafter (Fig. 3B). Nasal wash samples were collected every other day for 10 days after the virus challenge, and 3 ferrets were sacrificed at 3 and 6 days postinfection (dpi) to harvest the lungs. Consistently with the trends shown in body temperature and weight, immunized ferrets showed rapid viral clearance in the nasal washes (Fig. 3C) and lungs (Fig. 3D) of both groups of vaccinated ferrets. It should be noted that i.m. and i.n. immunization resulted in slightly more effective viral clearance in nasal washes at 4 dpi than i.m. immunization (Fig. 3C).

To further investigate the potency of protective immunity by RBD-nanoparticles, we challenged the immunized ferrets with a higher titer (10^6.0^ TCID_50_/ml) of SARS-CoV-2 by following the same immunization protocol (Fig. 2A). Consistently, RBD-nanoparticle-immunized ferrets showed no increase of body temperature compared to adjuvant-only-immunized ferrets (see Fig. S1 in the supplemental material). While adjuvant-only-immunized ferrets suffered from cough, runny nose, and reduction in movement, RBD-nanoparticle-immunized ferrets showed only a mild reduction in movement on the 2nd and 3rd days after the high-virus-titer challenge (Table 1). On the other hand, i.n. and i.m. immunization resulted in more potent protective immunity upon challenge with a high virus titer than i.n. immunization only (Fig. S2). Intranasal and i.m. immunization led to faster clearance of infectious virus in nasal washes at 4 and 8 dpi than i.m. immunization alone (Fig. S2A). Infectious virus titers of lungs were also lower in i.n. and i.m. immunized ferrets than in i.m. immunized ferrets (Fig. S2B). These data demonstrate that RBD-nanoparticles induce strong protective immunity to suppress SARS-CoV-2-induced clinical symptoms and promote viral clearance. Moreover, a combination of i.n. and i.m. immunization induces a stronger antiviral immunity against challenge with high-titer SARS-CoV-2 than i.m. immunization alone.

**TABLE 1:** RBD-nanoparticle immunization suppresses clinical symptoms induced by challenge with a high SARS-CoV-2 titer^*a*^

Group (*n* = 4/group)	Clinical symptom	No. of animals at dpi:									
		0	1	2	3	4	5	6	7	8	10
Adjuvant only	Cough	0	0	0.5^*b*^	1	1	0	0	0	0	0
	Runny nose	0	0	1.0	1	1	1	1	0.75	0	0
	Movement, activity	0	0	1.25	2	2	1.25	0.75	0.5	0	0
Total		0	0	2.75	4	4	2.25	1.75	1.25	0	0

RBD-nanoparticles	Cough	0	0	0	0	0	0	0	0	0	0
	Runny nose	0	0	0	0	0	0	0	0	0	0
	Movement, activity	0	0	0.75	0.5	0	0	0	0	0	0
Total		0	0	0.75	0.5	0	0	0	0	0	0

a A group of adjuvant-immunized or RBD-nanoparticle i.m. immunized ferrets were challenged with 10^6.0^ TCID_50_/ml of SARS-CoV-2 and observed for their clinical symptoms: cough, runny nose, movement, and activity. The symptoms were quantified as counts per 30 min. Score for cough: 0, no evidence of cough; 1, occasional cough; 2, frequent cough. For runny nose: 0, no nasal rattling or sneezing; 1, moderate nasal discharge on external nares; 2, severe nasal discharge on external nares. For movement and activity: 0, normal movement and activity; 1, mildly reduced movement and activity; 2, significantly reduced movement and activity.

b Average value of each ferret's clinical symptom score.

10.1128/mBio.00230-21.1 FIG S1 Body temperatures of RBD-nanoparticle-immunized ferrets after challenge with high-titer SARS-CoV-2. Body temperature changes of adjuvant-immunized, RBD-nanoparticle i.m. immunized, or RBD-nanoparticle i.m. and i.n. immunized ferrets upon high-titer SARS-CoV-2 challenge. Download FIG S1, PDF file, 0.02 MB. Copyright © 2021 Kim et al. 2021 Kim et al. https://creativecommons.org/licenses/by/4.0/ This is an open-access article distributed under the terms of the Creative Commons Attribution 4.0 International license.

10.1128/mBio.00230-21.2 FIG S2 Respiratory virus titer of RBD-nanoparticle-immunized ferrets after challenge with high-titer SARS-CoV-2. (A) Viral titers in nasal washes of adjuvant-immunized, RBD-nanoparticle i.m. immunized, or RBD-nanoparticle i.m. and i.n. immunized ferrets upon high-titer SARS-CoV-2 challenge. (B) Viral titers in lungs of adjuvant-immunized, RBD-nanoparticle i.m. immunized, or RBD-nanoparticle i.m. and i.n. immunized ferrets upon high-titer SARS-CoV-2 challenge. Infectious virus titers were measured and are shown as means ± standard errors of the means. Download FIG S2, PDF file, 0.03 MB. Copyright © 2021 Kim et al. 2021 Kim et al. https://creativecommons.org/licenses/by/4.0/ This is an open-access article distributed under the terms of the Creative Commons Attribution 4.0 International license.

### RBD-ferritin vaccination blocks lung damage from SARS-CoV-2 challenge.

**FIG 4:**
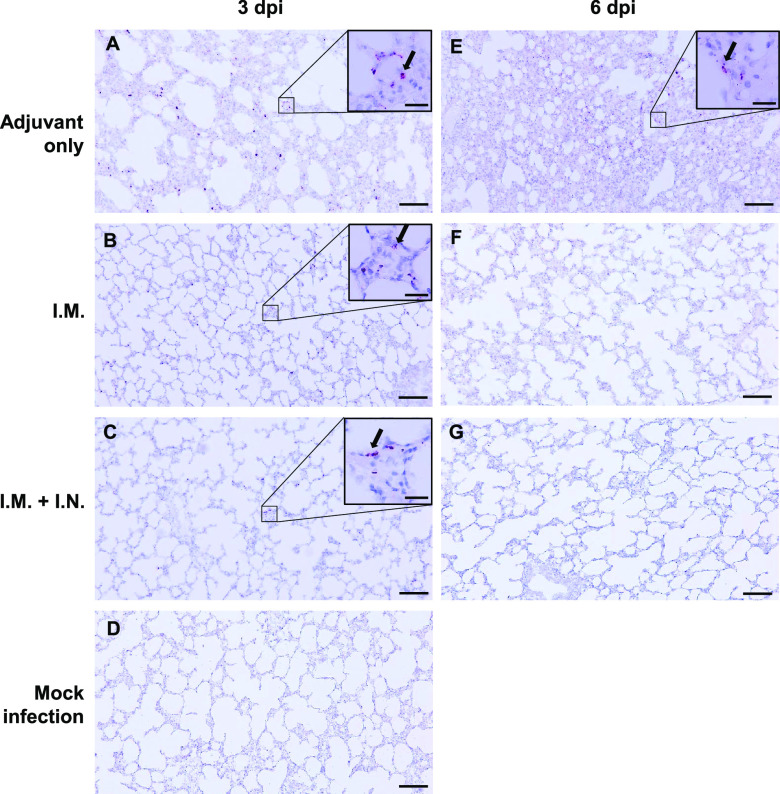
Lung histology and RNAscope results of immunized ferrets upon SARS-CoV-2 challenge. Adjuvant-immunized, RBD-nanoparticle i.m. immunized, or RBD-nanoparticle i.m. and i.n. immunized ferrets were intranasally inoculated with 10^5.0^ TCID_50_/ml of SARS-CoV-2. Tissues were harvested 3 and 6 dpi. RNAscope detected SARS-CoV-2 spike RNA-positive cells in lung tissues of adjuvant-immunized (A and E), RBD-nanoparticle i.m. immunized (B and F), and RBD-nanoparticle i.m. and i.n. immunized ferrets (C and G). Mock-infected ferret lung (D) was included as a control. The magnification is ×100, and scale bars represent 100 μm. Insets indicate the magnification (×400) of a SARS-CoV-2-positive image, and the scale bar represents 20 μm. Black arrows indicate SARS-CoV-2 RNA-positive cells.

COVID-19 has most commonly been shown to be associated with a spectrum of lung damage. To compare lung histopathologies among immunized ferrets, RNAscope *in situ* hybridization and histopathological examination were conducted (Fig. 4). Lung tissues harvested from naive ferrets were included as negative controls (Fig. 4D). RNAscope *in situ* hybridization results showed that the adjuvant-only-immunized ferrets had a number of SARS-CoV-2 RNA-positive cells at 3 and 6 dpi, with infiltration of numerous inflammatory immune cells (Fig. 4A to E). At 3 dpi, i.m. or i.m. and i.n. immunized ferrets showed considerable reduction of viral RNAs in the lungs compared to adjuvant-only-immunized ferrets (Fig. 4). At 6 dpi, lung tissues of i.m. or i.m. and i.n. immunized ferrets showed complete clearance of viral RNAs (Fig. 4F and G), while adjuvant-only-immunized ferrets still showed high viral RNAs (Fig. 4E). Finally, i.m. or i.m. and i.n. immunized ferrets showed little or no infiltration of inflammatory immune cells in infected lungs (Fig. 4B to G). These data show that RBD-nanoparticle immunization accelerates viral clearance in the lung and suppresses the infiltration of inflammatory immune cells.

## DISCUSSION

Since its first discovery in Wuhan, China, in late 2019, SARS-CoV-2 has rapidly spread around the world and was declared a pandemic in 3 months. Confirmed infection and death counts have skyrocketed to over 88 million infections and 2 million deaths, and the statistics are still on a continuous rise. Although 80% of the infections do not progress to severe COVID-19, the recent surge in infections and severe patients have led to subsequent increases in mortality rates (8, 9). While several vaccines were approved at accelerated rates (59, 60), additional in-depth study of mRNA-based vaccines regarding safety concerns and long-term effects still need to be addressed, as they are the first approved mRNA human vaccine of its kind. Moreover, taking the growing evidence of reinfections into consideration, recovered patients cannot be completely excluded from the population requiring vaccination (61–64). Therefore, there still is a constant need for alternative vaccine approaches against SARS-CoV-2 using relatively well-characterized approaches. Recent advances in nanotechnology has favorably allowed the application of nanoparticles in the field of vaccinology to develop safer, yet potent, vaccines. One of the most promising candidates is H. pylori-bullfrog ferritin, which has been genetically engineered to carry a protruding tail from the bullfrog on the self-assembling ferritin core of H. pylori and serves as a platform to build nanoparticles of immunogen. This approach has proven to have higher efficacy at a lower dose than traditional protein subunit vaccines. This approach also highlights the lower risk of vaccine-related adverse effects and potentially greater accessibility to the public with reduced production cost (38, 44, 45). Importantly, the inherent stability of ferritin nanoparticles from heat and chemicals may shed light on the removal of the necessity of the strict cold-chain supply required for the mRNA-based vaccines in current distribution (41).

SARS-CoV-2 carries the spike protein, which attaches to the host receptor ACE2, triggering membrane fusion for entry into host cells. The RBD of the spike protein confers the specificity to bind to ACE2 and therefore is a promising target for vaccine development throughout the *Coronaviridae* family. We selected the RBD as a vaccine antigen, chosen for previously developed vaccine candidates against coronaviruses (17, 65, 66). However, soluble antigen is weakly immunogenic and therefore requires a high dose of antigen along with an adjuvant, which correlates with a higher risk of vaccine-related adverse effects (29). In this study, we engineered the fusion of the SARS-CoV-2 spike RBD with H. pylori*-*bullfrog ferritin to develop an RBD-nanoparticle vaccine. Ferrets immunized with RBD-nanoparticles carried efficient neutralizing antibodies against SARS-CoV-2 and were protected from fever and body weight loss upon SARS-CoV-2 challenge. These clinical symptoms corresponded to the accelerated viral clearance in nasal washes and lungs following SARS-CoV-2 challenge. We further investigated the vaccine potential of RBD-nanoparticles by challenging the immunized ferrets with a high virus titer (10^6.0^ TCID_50_/ml). Immunized ferrets showed considerably reduced clinical symptoms, such as body weight loss, cough, runny nose, and movement activity, upon challenge with high-titer SARS-CoV-2. Moreover, RNAscope analyses showed rapid viral clearance in the lungs of immunized ferrets compared to clearance in adjuvant-only-immunized ferrets. Histological analysis also showed little or no lung tissue damage and inflammatory immune cell infiltration in immunized ferrets. As seen from other protein vaccines, such as the HPV VLP, which requires prime-boost regimens (67), the first immunization alone was not sufficient to induce neutralizing antibodies. i.n. plus i.m. immunization elicited more potent protective immunity upon challenge with high-titer SARS-CoV-2 than i.m. immunization alone, which is consistent with previous reports showing a stronger induction of mucosal immunity upon i.n. than i.m. immunization to protect against respiratory pathogens such as MERS-CoV (65, 66, 68), influenza virus (69), and Mycoplasma pneumoniae (70). To differentiate vaccine efficacy between i.n. immunization and i.n. and i.m. immunization, we repeated the viral challenge with a high titer (10^6.0^ TCID_50_/ml) and observed improvement in viral clearance in lung and nasal washes from i.n. and i.m. immunized ferrets. However, as i.n. and i.m. immunization was employed together in this study, further investigation is required to directly compare vaccine efficacies between i.n. immunization and i.m. immunization against SARS-CoV-2 infection. Also, ferrets challenged with a 10^6.0^ TCID_50_/ml virus titer showed delayed viral clearance compared to ferrets challenged with a 10^5.0^ TCID_50_/ml virus titer. However, 10^5.0^ TCID_50_/ml is already excessive and not physiologically relevant to a real clinical setting.

In this study, we integrated SARS-CoV-2-derived immunogen into self-assembling nanoparticles to develop an effective vaccine candidate against COVID-19. Intramuscularly immunized animals showed strong induction of neutralizing antibody, rapid clearance of respiratory track virus, and clear suppression of clinical symptoms, which is further enhanced in combination with intranasal immunization. However, additional comprehensive studies are needed to understand the humoral and cellular immunity elicited by RBD-nanoparticle administration and differential activation of IgA-mediated mucosal immunity by different immunization routes. Taken together, our study indicated that immunization with self-assembling SARS-CoV-2 RBD-nanoparticles elicits protective immunity against SARS-CoV-2 infection, showing its potential as a vaccine candidate in the midst of the COVID-19 pandemic.

## MATERIALS AND METHODS

### Materials and reagents.

See Table 2 for a list of materials and reagents.

**TABLE 2:** List of materials and reagents

Type of resource	Reagent or resource	Vendor or source	Catalog no.
Recombinant DNA	pFUSEN-hIgG1Fc	InvivoGen	pfcn-hg1
	H. pylori-bullfrog recombinant ferritin	Jefferey Cohen and Gary Nabel at the NIAID	
	SARS-CoV-2 spike (codon optimized for human codon usage)	GenScript	MC_0101081

Chemical	Polyethylene imine	Polysciences	23966
	Valproic acid	Sigma	P4543
	AddaVax adjuvant	InvivoGen	Vac-adx-10
	RNAscope reagent	ACD	322360
	RNAscope probe	ACD	848561
	Gill’s hematoxylin no. 1	Polysciences	24242

Purification	Labscale Tangential Flow Filtration (TFF) system	Sigma	C1975
	TFF filter (100-kDa MWCO)	Sigma	PXB100C50
	TFF filter (500-kDa MWCO)	Sigma	PXB500C50
	NGC medium-pressure liquid chromatography system	Bio-Rad	
	BioFrac Fraction collector	Bio-Rad	7410002
	Superdex 200 Increase 10/300 GL column	Cytiva	45-002-570
	HiPrep 16/60 Sephacryl S-500 HR column	Cytiva	28935606

### Expression vector construction.

The gene encoding the recombinant ferritin engineered from Helicobacter pylori nonheme ferritin and the 2nd to 9th residues of the bullfrog (*Rana catesbeiana*) ferritin lower subunit was a gift from Gary Nabel (44). The gene encoding spike of SARS-CoV-2 (GenBank accession no. NC_0101080), codon optimized for human codon usage (GenBank accession no. MC_0101081), was purchased from GenScript (pUC57-2019-nCoV-S). The RBD was used to generate a fragment encoding the RBD-SSGGASVLA linker-recombinant ferritin. For the expression plasmid, a commercially available pFUSE vector (InvivoGen) was engineered to replace the human ferritin light-chain gene promoter with the simian virus 40 (SV40) promoter. Genes encoding the recombinant ferritin and the RBD-linker-ferritin fragment were cloned into the plasmid vector.

### Computer-assisted three-dimensional model of nanoparticles.

Previously solved structures of the H. pylori ferritin nanoparticle (Protein Data Bank [PDB] accession no. 3EGM) and SARS-CoV-2 RBD (PDB accession no. 7JMP) were processed with PyMOL (Schrodinger) and Autodesk Meshmixer (Autodesk). The model was generated to reflect the linker connecting the end of the RBD to the start of H. pylori ferritin monomer.

### Expression and purification of nanoparticles.

HEK293T cells were directly purchased from the American Type Culture Collection (ATCC) and maintained in Dulbecco’s modified Eagle’s medium (DMEM; Gibco) supplemented with 10% fetal bovine serum (FBS; Gibco) and 1% penicillin/streptomycin (Gibco). The cells were transiently transfected with polyethylenimine (Polysciences) and respective vector plasmids in Opti-MEM and FreeStyle 293 medium (Gibco) supplemented with 3 mM valproic acid. Supernatants containing the nanoparticle were harvested 72 h after transfection and concentrated with the Labscale TFF system equipped with filters (Millipore Sigma) with 100-kDa and 500-kDa molecular weight cutoffs (MWCO). The concentrates were purified by size exclusion chromatography (NGC medium-pressure liquid chromatography; Bio-Rad) using Superdex 200 10/300 GL and HiPrep 16/60 Sephacryl S-500 HR (Cytiva) columns running degassed phosphate-buffered saline (PBS) at 0.4 ml/min. Standard curves were plotted using a gel filtration low-molecular-weight/high-molecular-weight (LMW/HMW) calibration kit (Cytiva) running at the same conditions. Collected fractions were verified for their yield and purity via SDS-PAGE and stored at −80°C in 10% glycerol (Invitrogen).

### Virus propagation.

The NMC2019-nCoV02 strain of SARS-CoV-2 was isolated from a patient diagnosed with COVID-19 who tested positive for SARS-CoV-2 in February 2020 in South Korea. Vero cells were used to propagate the virus in DMEM (Gibco) supplemented with 1% penicillin/streptomycin (Gibco) at 37°C. The viruses were harvested 72 h later and stored at −80°C until use.

### Animal care.

Male and female ferrets that were 16 to 20 months old and tested seronegative for influenza A virus, MERS-CoV, and SARS-CoV were purchased from ID Bio Corporation (Cheongju, South Korea). The ferrets were housed in an animal biosafety level 3 (ABSL3) facility within Chungbuk National University (Cheongju, South Korea) with a 12-h light/dark cycle and with access to water and diet. All animal care was performed strictly according to the animal care guidelines and experiment protocols approved by the Institutional Animal Care and Use Committee (IACUC) of Chungbuk National University.

### Ferret immunizations and viral challenge.

RBD-ferritin nanoparticles (volume, 300 μl) and AddaVax adjuvant (volume, 300 μl) were administered into the legs through intramuscular injection and/or the intranasal route. Subsequently, ferrets were intranasally infected with 10^5.0^ or 10^6.0^ TCID_50_/ml SARS-CoV-2. Body weight and temperature were measured, and veterinary clinical symptoms were observed every day. Blood and nasal washes were collected every other day for 10 days. Three animals per group were sacrificed at days 3 and 6 to collect lung tissues with individual scissors. Infectious viruses from the nasal washes and lung tissues were quantified by inoculation onto Vero cells. Veterinary symptoms were scored according to the procedure used in our previous publication (57).

### Titration of neutralizing antibody in serum.

The neutralizing antibody assay against SARS-CoV-2 was carried out using a microneutralization assay in Vero cells. Collected ferret serum specimens were inactivated at 56°C for 30 min. Initial 1:2 serum dilutions were made with the medium, and 2-fold serial dilutions of all samples were made to a final serum dilution of 1:2 to 1:256. For each well, 50 μl of serially diluted serum was mixed with 50 μl (equal volume) of 100 TCID_50_ of SARS-CoV-2 and incubated at 37°C for 1 h to neutralize the infectious virus. The mixtures were then transferred to Vero cell monolayers. Vero cells were incubated at 37°C in 5% CO_2_ for 4 days and monitored for a 50% reduction in cytopathic effect (CPE).

### RNAscope.

SARS-CoV-2 RNA (spike gene) was detected using the spike-specific probe (Advanced Cell Diagnostics; catalog [cat.] no. 848561) and visualized using an RNAscope 2.5 HD RED reagent kit (Advanced Cell Diagnostics; cat. no. 322360). Lung tissue sections were fixed in 4% neutral buffered formalin and embedded in paraffin, according to the manufacturer’s instructions, followed by counterstaining with 50% Gill’s hematoxylin no. 1 (Polysciences; cat. no. 24242-1000). Slides were viewed using an Olympus IX 71 (Olympus, Tokyo, Japan) microscope with DP controller software to capture images.

### Statistical analysis.

Asterisks in all figures indicate statistical significance compared with the adjuvant-only group as evaluated by the two-way analysis of variance (ANOVA) Dunnett multiple-comparison test (* indicates a *P* of <0.05, ** indicates a *P* of <0.01, *** indicates a *P* of <0.001, and **** indicates a *P* of <0.0001). Figures were drawn using GraphPad Prism 8 (GraphPad).

## References

[1] Zhu N, Zhang D, Wang W, Li X, Yang B, Song J, Zhao X, Huang B, Shi W, Lu R, Niu P, Zhan F, Ma X, Wang D, Xu W, Wu G, Gao GF, Tan W, China Novel Coronavirus Investigating and Research Team. 2020. A novel coronavirus from patients with pneumonia in China, 2019. N Engl J Med 382:727–733. doi:10.1056/NEJMoa2001017. 31978945 PMC7092803

[2] Coronaviridae Study Group of the International Committee on Taxonomy of Viruses. 2020. The species severe acute respiratory syndrome-related coronavirus: classifying 2019-nCoV and naming it SARS-CoV-2. Nat Microbiol 5:536–544. doi:10.1038/s41564-020-0695-z. 32123347 PMC7095448

[3] WHO. 2020. Statement on the second meeting of the International Health Regulations (2005) Emergency Committee regarding the outbreak of novel coronavirus (2019-nCoV). World Health Organization, Geneva, Switzerland. https://www.who.int/news/item/30-01-2020-statement-on-the-second-meeting-of-the-international-health-regulations-(2005)-emergency-committee-regarding-the-outbreak-of-novel-coronavirus-(2019-ncov). Accessed 11 January 2021.

[4] WHO. 2020. Coronavirus disease 2019 (COVID-19) situation report-63. World Health Organization, Geneva, Switzerland. https://www.who.int/docs/default-source/coronaviruse/situation-reports/20200323-sitrep-63-covid-19.pdf?sfvrsn=b617302d_4. Accessed 11 January 2021.

[5] GISAID. Coronavirus COVID-19 global cases by Johns Hopkins CSSE. GISAID, Munich, Germany. https://www.gisaid.org/epiflu-applications/global-cases-covid-19. Accessed 11 January 2021.

[6] Wu Z, McGoogan JM. 2020. Characteristics of and important lessons from the coronavirus disease 2019 (COVID-19) outbreak in China: summary of a report of 72314 cases from the Chinese Center for Disease Control and Prevention. JAMA 323:1239–1242. doi:10.1001/jama.2020.2648. 32091533

[7] NIH. 2020. COVID-19 treatment guidelines. NIH, Bethesda, MD. https://www.covid19treatmentguidelines.nih.gov/. Accessed 11 January 2021.

[8] Harpen NA, Tan KS. 2020. United States resource availability for COVID-19. Society of Critical Care Medicine, Mount Prospect, IL. https://www.sccm.org/Blog/March-2020/United-States-Resource-Availability-for-COVID-19.

[9] Ji Y, Ma Z, Peppelenbosch MP, Pan Q. 2020. Potential association between COVID-19 mortality and health-care resource availability. Lancet Glob Health 8:e480. doi:10.1016/S2214-109X(20)30068-1. 32109372 PMC7128131

[10] Yelin D, Wirtheim E, Vetter P, Kalil AC, Bruchfeld J, Runold M, Guaraldi G, Mussini C, Gudiol C, Pujol M, Bandera A, Scudeller L, Paul M, Kaiser L, Leibovici L. 2020. Long-term consequences of COVID-19: research needs. Lancet Infect Dis 20:1115–1117. doi:10.1016/S1473-3099(20)30701-5. 32888409 PMC7462626

[11] Del Rio C, Collins LF, Malani P. 2020. Long-term health consequences of COVID-19. JAMA 324:1723. doi:10.1001/jama.2020.19719. 33031513 PMC8019677

[12] Wu F, Zhao S, Yu B, Chen YM, Wang W, Song ZG, Hu Y, Tao ZW, Tian JH, Pei YY, Yuan ML, Zhang YL, Dai FH, Liu Y, Wang QM, Zheng JJ, Xu L, Holmes EC, Zhang YZ. 2020. A new coronavirus associated with human respiratory disease in China. Nature 579:265–269. doi:10.1038/s41586-020-2008-3. 32015508 PMC7094943

[13] Chan JF, Kok KH, Zhu Z, Chu H, To KK, Yuan S, Yuen KY. 2020. Genomic characterization of the 2019 novel human-pathogenic coronavirus isolated from a patient with atypical pneumonia after visiting Wuhan. Emerg Microbes Infect 9:221–236. doi:10.1080/22221751.2020.1719902. 31987001 PMC7067204

[14] Cai Y, Zhang J, Xiao T, Peng H, Sterling SM, Walsh RM, Jr, Rawson S, Rits-Volloch S, Chen B. 2020. Distinct conformational states of SARS-CoV-2 spike protein. Science 369:1586–1592. doi:10.1126/science.abd4251. 32694201 PMC7464562

[15] Kim YS, Son A, Kim J, Kwon SB, Kim MH, Kim P, Kim J, Byun YH, Sung J, Lee J, Yu JE, Park C, Kim YS, Cho NH, Chang J, Seong BL. 2018. Chaperna-mediated assembly of ferritin-based Middle East respiratory syndrome-coronavirus nanoparticles. Front Immunol 9:1093. doi:10.3389/fimmu.2018.01093. 29868035 PMC5966535

[16] He Y, Zhou Y, Liu S, Kou Z, Li W, Farzan M, Jiang S. 2004. Receptor-binding domain of SARS-CoV spike protein induces highly potent neutralizing antibodies: implication for developing subunit vaccine. Biochem Biophys Res Commun 324:773–781. doi:10.1016/j.bbrc.2004.09.106. 15474494 PMC7092904

[17] Jiang S, Lu L, Liu Q, Xu W, Du L. 2012. Receptor-binding domains of spike proteins of emerging or re-emerging viruses as targets for development of antiviral vaccines. Emerg Microbes Infect 1:e13. doi:10.1038/emi.2012.1. 26038424 PMC3630917

[18] Pallesen J, Wang N, Corbett KS, Wrapp D, Kirchdoerfer RN, Turner HL, Cottrell CA, Becker MM, Wang L, Shi W, Kong WP, Andres EL, Kettenbach AN, Denison MR, Chappell JD, Graham BS, Ward AB, McLellan JS. 2017. Immunogenicity and structures of a rationally designed prefusion MERS-CoV spike antigen. Proc Natl Acad Sci U S A 114:E7348–E7357. doi:10.1073/pnas.1707304114. 28807998 PMC5584442

[19] Okba NMA, Muller MA, Li W, Wang C, GeurtsvanKessel CH, Corman VM, Lamers MM, Sikkema RS, de Bruin E, Chandler FD, Yazdanpanah Y, Le Hingrat Q, Descamps D, Houhou-Fidouh N, Reusken C, Bosch BJ, Drosten C, Koopmans MPG, Haagmans BL. 2020. Severe acute respiratory syndrome coronavirus 2-specific antibody responses in coronavirus disease patients. Emerg Infect Dis 26:1478–1488. doi:10.3201/eid2607.200841. 32267220 PMC7323511

[20] Liu L, Wang P, Nair MS, Yu J, Rapp M, Wang Q, Luo Y, Chan JF, Sahi V, Figueroa A, Guo XV, Cerutti G, Bimela J, Gorman J, Zhou T, Chen Z, Yuen KY, Kwong PD, Sodroski JG, Yin MT, Sheng Z, Huang Y, Shapiro L, Ho DD. 2020. Potent neutralizing antibodies against multiple epitopes on SARS-CoV-2 spike. Nature 584:450–456. doi:10.1038/s41586-020-2571-7. 32698192

[21] Liu B, Shi Y, Zhang W, Li R, He Z, Yang X, Pan Y, Deng X, Tan M, Zhao L, Zou F, Zhang Y, Pan T, Zhang J, Zhang X, Xiao F, Li F, Deng K, Zhang H. 2020. Recovered COVID-19 patients with recurrent viral RNA exhibit lower levels of anti-RBD antibodies. Cell Mol Immunol 17:1098–1100. doi:10.1038/s41423-020-00528-0. 32939033 PMC7493297

[22] Piccoli L, Park YJ, Tortorici MA, Czudnochowski N, Walls AC, Beltramello M, Silacci-Fregni C, Pinto D, Rosen LE, Bowen JE, Acton OJ, Jaconi S, Guarino B, Minola A, Zatta F, Sprugasci N, Bassi J, Peter A, De Marco A, Nix JC, Mele F, Jovic S, Rodriguez BF, Gupta SV, Jin F, Piumatti G, Lo Presti G, Pellanda AF, Biggiogero M, Tarkowski M, Pizzuto MS, Cameroni E, Havenar-Daughton C, Smithey M, Hong D, Lepori V, Albanese E, Ceschi A, Bernasconi E, Elzi L, Ferrari P, Garzoni C, Riva A, Snell G, Sallusto F, Fink K, Virgin HW, Lanzavecchia A, Corti D, Veesler D. 2020. Mapping neutralizing and immunodominant sites on the SARS-CoV-2 spike receptor-binding domain by structure-guided high-resolution serology. Cell 183:1024–1042.e21. doi:10.1016/j.cell.2020.09.037. 32991844 PMC7494283

[23] Wu NC, Yuan M, Liu H, Lee CD, Zhu X, Bangaru S, Torres JL, Caniels TG, Brouwer PJM, van Gils MJ, Sanders RW, Ward AB, Wilson IA. 2020. An alternative binding mode of IGHV3-53 antibodies to the SARS-CoV-2 receptor binding domain. Cell Rep 33:108274. doi:10.1016/j.celrep.2020.108274. 33027617 PMC7522650

[24] Walsh EE, Frenck RW, Falsey AR, Kitchin N, Absalon J, Gurtman A, Lockhart S, Neuzil K, Mulligan MJ, Bailey R, Swanson KA, Li P, Koury K, Kalina W, Cooper D, Fontes-Garfias C, Shi P-Y, Türeci Ö, Tompkins KR, Lyke KE, Raabe V, Dormitzer PR, Jansen KU, Şahin U, Gruber WC. 2020. Safety and immunogenicity of two RNA-based Covid-19 vaccine candidates. N Engl J Med 383:2439–2450. doi:10.1056/NEJMoa2027906. 33053279 PMC7583697

[25] Baden LR, El Sahly HM, Essink B, Kotloff K, Frey S, Novak R, Diemert D, Spector SA, Rouphael N, Creech CB, McGettigan J, Kehtan S, Segall N, Solis J, Brosz A, Fierro C, Schwartz H, Neuzil K, Corey L, Gilbert P, Janes H, Follmann D, Marovich M, Mascola J, Polakowski L, Ledgerwood J, Graham BS, Bennett H, Pajon R, Knightly C, Leav B, Deng W, Zhou H, Han S, Ivarsson M, Miller J, Zaks T, for the COVE Study Group. 2021. Efficacy and safety of the mRNA-1273 SARS-CoV-2 vaccine. N Engl J Med 384:403–416. doi:10.1056/NEJMoa2035389. 33378609 PMC7787219

[26] Sadoff J, De Paepe E, Haazen W, Omoruyi E, Bastian AR, Comeaux C, Heijnen E, Strout C, Schuitemaker H, Callendret B. 26 8 2020. Safety and immunogenicity of the Ad26.RSV.preF investigational vaccine coadministered with an influenza vaccine in older adults. J Infect Dis doi:10.1093/infdis/jiaa409. 32851411

[27] Keech C, Albert G, Cho I, Robertson A, Reed P, Neal S, Plested JS, Zhu M, Cloney-Clark S, Zhou H, Smith G, Patel N, Frieman MB, Haupt RE, Logue J, McGrath M, Weston S, Piedra PA, Desai C, Callahan K, Lewis M, Price-Abbott P, Formica N, Shinde V, Fries L, Lickliter JD, Griffin P, Wilkinson B, Glenn GM. 2020. Phase 1–2 trial of a SARS-CoV-2 recombinant spike protein nanoparticle vaccine. N Engl J Med 383:2320–2332. doi:10.1056/NEJMoa2026920. 32877576 PMC7494251

[28] Cohen AA, Gnanapragasam PNP, Lee YE, Hoffman PR, Ou S, Kakutani LM, Keeffe JR, Wu HJ, Howarth M, West AP, Barnes CO, Nussenzweig MC, Bjorkman PJ. 12 1 2021. Mosaic nanoparticles elicit cross-reactive immune responses to zoonotic coronaviruses in mice. Science doi:10.1126/science.abf6840. PMC7928838 33436524

[29] Gause KT, Wheatley AK, Cui J, Yan Y, Kent SJ, Caruso F. 2017. Immunological principles guiding the rational design of particles for vaccine delivery. ACS Nano 11:54–68. doi:10.1021/acsnano.6b07343. 28075558

[30] Link A, Zabel F, Schnetzler Y, Titz A, Brombacher F, Bachmann MF. 2012. Innate immunity mediates follicular transport of particulate but not soluble protein antigen. J Immunol 188:3724–3733. doi:10.4049/jimmunol.1103312. 22427639

[31] Roldão A, Mellado MCM, Castilho LR, Carrondo MJT, Alves PM. 2010. Virus-like particles in vaccine development. Expert Rev Vaccines 9:1149–1176. doi:10.1586/erv.10.115. 20923267

[32] Qian C, Liu X, Xu Q, Wang Z, Chen J, Li T, Zheng Q, Yu H, Gu Y, Li S, Xia N. 2020. Recent progress on the versatility of virus-like particles. Vaccines (Basel) 8:139. doi:10.3390/vaccines8010139. PMC7157238 32244935

[33] Donaldson B, Lateef Z, Walker GF, Young SL, Ward VK. 2018. Virus-like particle vaccines: immunology and formulation for clinical translation. Expert Rev Vaccines 17:833–849. doi:10.1080/14760584.2018.1516552. 30173619 PMC7103734

[34] Ingale J, Stano A, Guenaga J, Sharma SK, Nemazee D, Zwick MB, Wyatt RT. 2016. High-density array of well-ordered HIV-1 spikes on synthetic liposomal nanoparticles efficiently activate B cells. Cell Rep 15:1986–1999. doi:10.1016/j.celrep.2016.04.078. 27210756 PMC4889521

[35] Thompson EA, Ols S, Miura K, Rausch K, Narum DL, Spångberg M, Juraska M, Wille-Reece U, Weiner A, Howard RF, Long CA, Duffy PE, Johnston L, O’Neil CP, Loré K. 2018. TLR-adjuvanted nanoparticle vaccines differentially influence the quality and longevity of responses to malaria antigen Pfs25. JCI Insight 3:e120692. doi:10.1172/jci.insight.120692. PMC6012510 29769448

[36] Marcandalli J, Fiala B, Ols S, Perotti M, de van der Schueren W, Snijder J, Hodge E, Benhaim M, Ravichandran R, Carter L, Sheffler W, Brunner L, Lawrenz M, Dubois P, Lanzavecchia A, Sallusto F, Lee KK, Veesler D, Correnti CE, Stewart LJ, Baker D, Lore K, Perez L, King NP. 2019. Induction of potent neutralizing antibody responses by a designed protein nanoparticle vaccine for respiratory syncytial virus. Cell 176:1420–1431.e17. doi:10.1016/j.cell.2019.01.046. 30849373 PMC6424820

[37] Yu F, Wang J, Dou J, Yang H, He X, Xu W, Zhang Y, Hu K, Gu N. 2012. Nanoparticle-based adjuvant for enhanced protective efficacy of DNA vaccine Ag85A-ESAT-6-IL-21 against Mycobacterium tuberculosis infection. Nanomedicine 8:1337–1344. doi:10.1016/j.nano.2012.02.015. 22406425

[38] Kanekiyo M, Wei CJ, Yassine HM, McTamney PM, Boyington JC, Whittle JR, Rao SS, Kong WP, Wang L, Nabel GJ. 2013. Self-assembling influenza nanoparticle vaccines elicit broadly neutralizing H1N1 antibodies. Nature 499:102–106. doi:10.1038/nature12202. 23698367 PMC8312026

[39] Hu X, Deng Y, Chen X, Zhou Y, Zhang H, Wu H, Yang S, Chen F, Zhou Z, Wang M, Qiu Z, Liao Y. 2017. Immune response of a novel ATR-AP205-001 conjugate anti-hypertensive vaccine. Sci Rep 7:12580. doi:10.1038/s41598-017-12996-y. 28974760 PMC5626684

[40] Pardi N, Hogan MJ, Naradikian MS, Parkhouse K, Cain DW, Jones L, Moody MA, Verkerke HP, Myles A, Willis E, LaBranche CC, Montefiori DC, Lobby JL, Saunders KO, Liao HX, Korber BT, Sutherland LL, Scearce RM, Hraber PT, Tombacz I, Muramatsu H, Ni H, Balikov DA, Li C, Mui BL, Tam YK, Krammer F, Kariko K, Polacino P, Eisenlohr LC, Madden TD, Hope MJ, Lewis MG, Lee KK, Hu SL, Hensley SE, Cancro MP, Haynes BF, Weissman D. 2018. Nucleoside-modified mRNA vaccines induce potent T follicular helper and germinal center B cell responses. J Exp Med 215:1571–1588. doi:10.1084/jem.20171450. 29739835 PMC5987916

[41] He D, Marles-Wright J. 2015. Ferritin family proteins and their use in bionanotechnology. N Biotechnol 32:651–657. doi:10.1016/j.nbt.2014.12.006. 25573765 PMC4571993

[42] Cho KJ, Shin HJ, Lee JH, Kim KJ, Park SS, Lee Y, Lee C, Park SS, Kim KH. 2009. The crystal structure of ferritin from Helicobacter pylori reveals unusual conformational changes for iron uptake. J Mol Biol 390:83–98. doi:10.1016/j.jmb.2009.04.078. 19427319

[43] Brito C, Matias C, Gonzalez-Nilo FD, Watt RK, Yevenes A. 2014. The C-terminal regions have an important role in the activity of the ferroxidase center and the stability of Chlorobium tepidum ferritin. Protein J 33:211–220. doi:10.1007/s10930-014-9552-3. 24609571

[44] Kanekiyo M, Bu W, Joyce MG, Meng G, Whittle JR, Baxa U, Yamamoto T, Narpala S, Todd JP, Rao SS, McDermott AB, Koup RA, Rossmann MG, Mascola JR, Graham BS, Cohen JI, Nabel GJ. 2015. Rational design of an Epstein-Barr virus vaccine targeting the receptor-binding site. Cell 162:1090–1100. doi:10.1016/j.cell.2015.07.043. 26279189 PMC4757492

[45] Kelly HG, Tan HX, Juno JA, Esterbauer R, Ju Y, Jiang W, Wimmer VC, Duckworth BC, Groom JR, Caruso F, Kanekiyo M, Kent SJ, Wheatley AK. 2020. Self-assembling influenza nanoparticle vaccines drive extended germinal center activity and memory B cell maturation. JCI Insight 5:e136653. doi:10.1172/jci.insight.136653. PMC7259527 32434990

[46] Chan KF, Carolan LA, Druce J, Chappell K, Watterson D, Young P, Korenkov D, Subbarao K, Barr IG, Laurie KL, Reading PC. 2018. Pathogenesis, humoral immune responses, and transmission between cohoused animals in a ferret model of human respiratory syncytial virus infection. J Virol 92:e01322-17. doi:10.1128/JVI.01322-17. 29187546 PMC5790937

[47] Oh DY, Lowther S, McCaw JM, Sullivan SG, Leang SK, Haining J, Arkinstall R, Kelso A, McVernon J, Barr IG, Middleton D, Hurt AC. 2014. Evaluation of oseltamivir prophylaxis regimens for reducing influenza virus infection, transmission and disease severity in a ferret model of household contact. J Antimicrob Chemother 69:2458–2469. doi:10.1093/jac/dku146. 24840623 PMC4130381

[48] Chan KF, Carolan LA, Korenkov D, Druce J, McCaw J, Reading PC, Barr IG, Laurie KL. 2018. Investigating viral interference between influenza A virus and human respiratory syncytial virus in a ferret model of infection. J Infect Dis 218:406–417. doi:10.1093/infdis/jiy184. 29746640 PMC7107400

[49] Cameron MJ, Kelvin AA, Leon AJ, Cameron CM, Ran L, Xu L, Chu YK, Danesh A, Fang Y, Li Q, Anderson A, Couch RC, Paquette SG, Fomukong NG, Kistner O, Lauchart M, Rowe T, Harrod KS, Jonsson CB, Kelvin DJ. 2012. Lack of innate interferon responses during SARS coronavirus infection in a vaccination and reinfection ferret model. PLoS One 7:e45842. doi:10.1371/journal.pone.0045842. 23029269 PMC3454321

[50] Chu YK, Ali GD, Jia F, Li Q, Kelvin D, Couch RC, Harrod KS, Hutt JA, Cameron C, Weiss SR, Jonsson CB. 2008. The SARS-CoV ferret model in an infection-challenge study. Virology 374:151–163. doi:10.1016/j.virol.2007.12.032. 18234270 PMC2831213

[51] Enkirch T, von Messling V. 2015. Ferret models of viral pathogenesis. Virology 479-480:259–270. doi:10.1016/j.virol.2015.03.017. 25816764 PMC7111696

[52] Johnson-Delaney CA, Orosz SE. 2011. Ferret respiratory system: clinical anatomy, physiology, and disease. Vet Clin North Am Exot Anim Pract 14:357–367, vii. doi:10.1016/j.cvex.2011.03.001. 21601818

[53] Schlottau K, Rissmann M, Graaf A, Schon J, Sehl J, Wylezich C, Hoper D, Mettenleiter TC, Balkema-Buschmann A, Harder T, Grund C, Hoffmann D, Breithaupt A, Beer M. 2020. SARS-CoV-2 in fruit bats, ferrets, pigs, and chickens: an experimental transmission study. Lancet Microbe 1:e218–e225. doi:10.1016/S2666-5247(20)30089-6. 32838346 PMC7340389

[54] Shi J, Wen Z, Zhong G, Yang H, Wang C, Huang B, Liu R, He X, Shuai L, Sun Z, Zhao Y, Liu P, Liang L, Cui P, Wang J, Zhang X, Guan Y, Tan W, Wu G, Chen H, Bu Z. 2020. Susceptibility of ferrets, cats, dogs, and other domesticated animals to SARS-coronavirus 2. Science 368:1016–1020. doi:10.1126/science.abb7015. 32269068 PMC7164390

[55] Kim YI, Kim SG, Kim SM, Kim EH, Park SJ, Yu KM, Chang JH, Kim EJ, Lee S, Casel MAB, Um J, Song MS, Jeong HW, Lai VD, Kim Y, Chin BS, Park JS, Chung KH, Foo SS, Poo H, Mo IP, Lee OJ, Webby RJ, Jung JU, Choi YK. 2020. Infection and rapid transmission of SARS-CoV-2 in ferrets. Cell Host Microbe 27:704–709.e2. doi:10.1016/j.chom.2020.03.023. 32259477 PMC7144857

[56] Ryan KA, Bewley KR, Fotheringham SA, Slack GS, Brown P, Hall Y, Wand NI, Marriott AC, Cavell BE, Tree JA, Allen L, Aram MJ, Bean TJ, Brunt E, Buttigieg KR, Carter DP, Cobb R, Coombes NS, Findlay-Wilson SJ, Godwin KJ, Gooch KE, Gouriet J, Halkerston R, Harris DJ, Hender TH, Humphries HE, Hunter L, Ho CMK, Kennard CL, Leung S, Longet S, Ngabo D, Osman KL, Paterson J, Penn EJ, Pullan ST, Rayner E, Skinner O, Steeds K, Taylor I, Tipton T, Thomas S, Turner C, Watson RJ, Wiblin NR, Charlton S, Hallis B, Hiscox JA, Funnell S, Dennis MJ, . 2021. Dose-dependent response to infection with SARS-CoV-2 in the ferret model and evidence of protective immunity. Nat Commun 12:81. doi:10.1038/s41467-020-20439-y. 33398055 PMC7782478

[57] Park SJ, Yu KM, Kim YI, Kim SM, Kim EH, Kim SG, Kim EJ, Casel MAB, Rollon R, Jang SG, Lee MH, Chang JH, Song MS, Jeong HW, Choi Y, Chen W, Shin WJ, Jung JU, Choi YK. 2020. Antiviral efficacies of FDA-approved drugs against SARS-CoV-2 infection in ferrets. mBio 11 doi:10.1128/mBio.01114-20. PMC7244896 32444382

[58] Holmgren J, Czerkinsky C. 2005. Mucosal immunity and vaccines. Nat Med 11:S45–S53. doi:10.1038/nm1213. 15812489

[59] FDA. 2020. Pfizer-BioNTech COVID-19 vaccine (BNT162, PF-07302048) vaccines and related biological products advisory committee briefing document. FDA, Silver Spring, MD.

[60] FDA. 2020. MRNA-1273 vaccines and related biological products advisory committee. FDA, Silver Spring, MD.

[61] Tillett RL, Sevinsky JR, Hartley PD, Kerwin H, Crawford N, Gorzalski A, Laverdure C, Verma SC, Rossetto CC, Jackson D, Farrell MJ, Van Hooser S, Pandori M. 2021. Genomic evidence for reinfection with SARS-CoV-2: a case study. Lancet Infect Dis 21:52–58. doi:10.1016/S1473-3099(20)30764-7. 33058797 PMC7550103

[62] To KK, Hung IF, Ip JD, Chu AW, Chan WM, Tam AR, Fong CH, Yuan S, Tsoi HW, Ng AC, Lee LL, Wan P, Tso E, To WK, Tsang D, Chan KH, Huang JD, Kok KH, Cheng VC, Yuen KY. 25 8 2020. COVID-19 re-infection by a phylogenetically distinct SARS-coronavirus-2 strain confirmed by whole genome sequencing. Clin Infect Dis doi:10.1093/cid/ciaa1275. PMC7499500 32840608

[63] Gupta V, Bhoyar RC, Jain A, Srivastava S, Upadhayay R, Imran M, Jolly B, Divakar MK, Sharma D, Sehgal P, Ranjan G, Gupta R, Scaria V, Sivasubbu S. 23 9 2020. Asymptomatic reinfection in two healthcare workers from India with genetically distinct SARS-CoV-2. Clin Infect Dis doi:10.1093/cid/ciaa1451. PMC7543380 32964927

[64] Volz E, Hill V, McCrone JT, Price A, Jorgensen D, O'Toole A, Southgate J, Johnson R, Jackson B, Nascimento FF, Rey SM, Nicholls SM, Colquhoun RM, da Silva Filipe A, Shepherd J, Pascall DJ, Shah R, Jesudason N, Li K, Jarrett R, Pacchiarini N, Bull M, Geidelberg L, Siveroni I, Consortium C-U, Goodfellow I, Loman NJ, Pybus OG, Robertson DL, Thomson EC, Rambaut A, Connor TR, COG-UK Consortium. 2021. Evaluating the effects of SARS-CoV-2 spike mutation D614G on transmissibility and pathogenicity. Cell 184:64–75.e11. doi:10.1016/j.cell.2020.11.020. 33275900 PMC7674007

[65] Kim MH, Kim HJ, Chang J. 2019. Superior immune responses induced by intranasal immunization with recombinant adenovirus-based vaccine expressing full-length spike protein of Middle East respiratory syndrome coronavirus. PLoS One 14:e0220196. doi:10.1371/journal.pone.0220196. 31329652 PMC6645677

[66] Ma C, Li Y, Wang L, Zhao G, Tao X, Tseng CT, Zhou Y, Du L, Jiang S. 2014. Intranasal vaccination with recombinant receptor-binding domain of MERS-CoV spike protein induces much stronger local mucosal immune responses than subcutaneous immunization: implication for designing novel mucosal MERS vaccines. Vaccine 32:2100–2108. doi:10.1016/j.vaccine.2014.02.004. 24560617 PMC4194189

[67] Sankaranarayanan R, Prabhu PR, Pawlita M, Gheit T, Bhatla N, Muwonge R, Nene BM, Esmy PO, Joshi S, Poli UR, Jivarajani P, Verma Y, Zomawia E, Siddiqi M, Shastri SS, Jayant K, Malvi SG, Lucas E, Michel A, Butt J, Vijayamma JM, Sankaran S, Kannan TP, Varghese R, Divate U, Thomas S, Joshi G, Willhauck-Fleckenstein M, Waterboer T, Muller M, Sehr P, Hingmire S, Kriplani A, Mishra G, Pimple S, Jadhav R, Sauvaget C, Tommasino M, Pillai MR, Indian HPV Vaccine Study Group. 2016. Immunogenicity and HPV infection after one, two, and three doses of quadrivalent HPV vaccine in girls in India: a multicentre prospective cohort study. Lancet Oncol 17:67–77. doi:10.1016/S1470-2045(15)00414-3. 26652797 PMC5357737

[68] Baz M, Samant M, Zekki H, Tribout-Jover P, Plante M, Lanteigne AM, Hamelin ME, Mallett C, Papadopoulou B, Boivin G. 2012. Effects of different adjuvants in the context of intramuscular and intranasal routes on humoral and cellular immune responses induced by detergent-split A/H3N2 influenza vaccines in mice. Clin Vaccine Immunol 19:209–218. doi:10.1128/CVI.05441-11. 22190392 PMC3272927

[69] Muszkat M, Greenbaum E, Ben-Yehuda A, Oster M, Yeu'l E, Heimann S, Levy R, Friedman G, Zakay-Rones Z. 2003. Local and systemic immune response in nursing-home elderly following intranasal or intramuscular immunization with inactivated influenza vaccine. Vaccine 21:1180–1186. doi:10.1016/s0264-410x(02)00481-4. 12559796

[70] Zhu C, Wu Y, Chen S, Yu M, Zeng Y, You X, Xiao J, Wang S. 2012. Protective immune responses in mice induced by intramuscular and intranasal immunization with a Mycoplasma pneumoniae P1C DNA vaccine. Can J Microbiol 58:644–652. doi:10.1139/w2012-041. 22540220

