# Supplementary material for: Development of Spike Receptor-Binding Domain Nanoparticles as a Vaccine Candidate against SARS-CoV-2 Infection in Ferrets
Source: mBio. 2021 Mar 2;12(2):e00230-21. doi: 10.1128/mBio.00230-21 (PMC8092224; doi:10.1128/mBio.00230-21)

Fig S2. Respiratory virus titer of RBD-nanoparticle immunized ferrets against challenge with high titer SARS-CoV-2

A. Viral titer in nasal washes

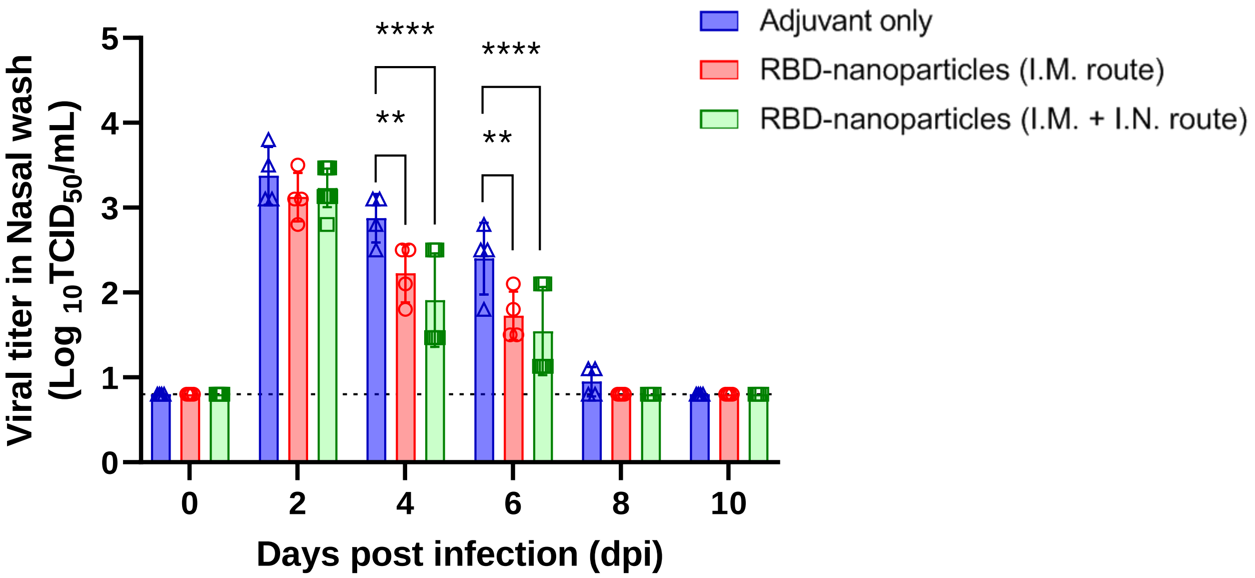

B. Viral titer in lungs

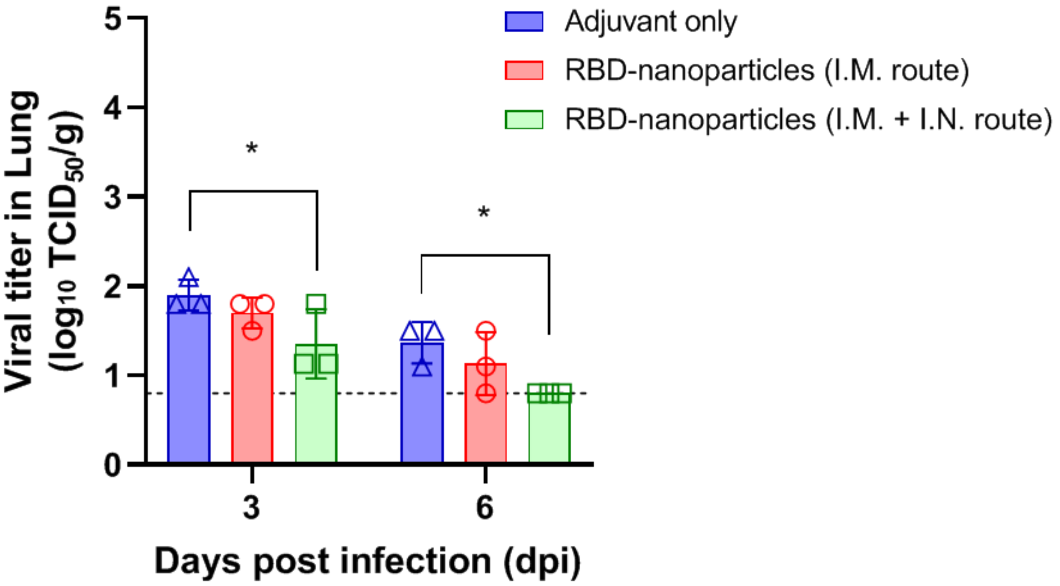

Supplement: FIG S2 [file mBio.00230-21-sf002.pdf]
